# Association of *CFH*, *LOC387715*, and *HTRA1* polymorphisms with exudative age-related macular degeneration in a northern Chinese population

**Published:** 2008-07-28

**Authors:** Yule Xu, Ning Guan, Jun Xu, Xiufen Yang, Kai Ma, Haiying Zhou, Feng Zhang, Torkel Snellingen, Yiqun Jiao, Xipu Liu, Ningli Wang, Ningpu Liu

**Affiliations:** 1 Beijing Tongren Eye Center, Capital Medical University, Beijing, China; 2 Beijing Sekwa Eye Hospital, Beijing, China

## Abstract

**Purpose:**

Variants in complement factor H (*CFH*), the hypothetical *LOC387715,* and the high-temperature requirement A-1 *(HTRA1)* genes have been reported to be associated with age-related macular degeneration (AMD). The purpose of this study was to investigate the association of reported common single-nucleotide polymorphisms (SNPs) in *CFH*, *LOC387715*, and *HTRA1* with exudative AMD in a northern Chinese population.

**Methods:**

A cohort of 121 unrelated patients with exudative AMD and 132 control subjects were enrolled in this study. Genomic DNA was extracted from blood leukocytes. Genotyping for SNPs rs1061170:T>C in *CFH* (Y402H), rs10490924:G>T in *LOC387715* (A69S), and rs11200638:G>A in the promoter of *HTRA1* was performed using a polymerase chain reaction (PCR) method followed by allele-specific restriction enzyme digestion and direct sequencing.

**Results:**

The Y402H variant in *CFH* was not associated with exudative AMD in our study population. Frequencies of Y402H was 10.3% in AMD cases and 8.0% in controls (p=0.353). Significant associations were detected for exudative AMD with SNPs rs10490924:G>T in *LOC387715* (A69S), and rs11200638:G>A in the promoter of *HTRA1*. The risk T-allele frequency of rs10490924 in *LOC387715* was 64.9% in cases versus 43.2% in controls (p<0.001). The odds ratio for risk of AMD was 1.56 (95% CI; 0.80–3.03) for the GT genotype and 5.45 (95% CI; 2.59–11.49) for the TT genotype. The A allele frequency of rs11200638 in the *HTRA1* promoter was 67.8% in cases versus 42.4% in controls (p<0.001). The odds ratio was 2.75 (95% CI; 1.34–5.64) for the GA genotype and 7.90 (95% CI; 3.61–17.26) for the AA genotype. An odds ratio of 7.94 (95% CI; 3.49–18.04) was obtained for carriers with both TT genotype in *LOC387715* and AA genotype in the *HTRA1* promoter.

**Conclusions:**

Our data suggest that the *LOC387715* and *HTRA1* polymorphisms are associated with a higher risk of exudative AMD in northern Chinese. We found no association of *CFH* Y402H with exudative AMD. The low frequency of *CFH* Y402H variant was further confirmed in this study population.

## Introduction

Age-related macular degeneration (AMD) is a clinically heterogeneous disease and the leading cause of irreversible visual impairment in the elderly population worldwide [1,2]. The early stage of the disease (referred to as age-related maculopathy) is characterized by the presence of drusen with areas of hyperpigmentation or depigmentation. As the disease progresses, two types of late stage AMD develop. The nonexudative AMD (dry or atrophic type) manifests as geographic atrophy or sharply demarcated area of depigmentation caused by atrophy of the retinal pigment epithelium (RPE) and overlying photoreceptors. Exudative AMD (wet or neovascular type) occurs when new blood vessels grow under the RPE or between RPE and neurosensory retina, leading to subretinal hemorrhage and subsequent scar tissue formation. Both nonexudative and exudative AMD result in the loss of central vision; however, nearly 90% of AMD cases with severe vision loss have exudative form of AMD. The Beijing Eye Study revealed that in Chinese older than 40 years, 2% of low vision cases and 7.7% of blindness cases were caused by AMD [3]. As this population ages, these numbers will likely rise.

AMD susceptibility is linked to both genetic and environmental factors, although its precise etiology remains elusive. Reported risk factors include ocular pigmentation, dietary factors, positive family history for AMD, smoking, and several gene mutations such as ATP-binding cassette transporter protein 4 (*ABCA4*), apolipoprotein E (*APOE*), and fibulin-5 (*FBLN5*) [4-9]. Moreover, genome-wide linkage studies have successfully identified several major chromosomal regions including 1q31 and 10q26 [10-14].

Recently, the complement factor H (*CFH*) gene on chromosome 1q31 has been demonstrated as the first major AMD susceptibility gene, and may associate with 30%–50% of AMD cases [15-18]. The Y402H variant in exon 9 (rs1061170:T>C) and other intron variants in *CFH* have been reported to be associated with an increased risk of AMD in more than ten different populations of European descent [15-23]. Susceptibility for AMD at putative genomic locus *LOC387715* on chromosome 10q26 was further confirmed in this group [24,25]. The strongest association centered over a frequent coding single-nucleotide polymorphism (SNP), rs10490924:G>T in exon 1 of *LOC387715* (A69S), strongly implicating this gene in the pathogenesis of AMD. Furthermore, studies of Hong Kong Chinese and Caucasian population have identified SNP rs11200638:G>A in the promoter of high-temperature requirement A-1 (*HTRA1*) gene, approximately 6.1 kb downstream of rs10490924 in *LOC387715* on chromosome 10q26, to be associated with increased risk of AMD [26,27].

It is critical to conduct studies in different populations in order to draw firm conclusions about the role of genetic factors. The purpose of this study was to investigate whether the reported major risk alleles in the *CFH*, *LOC387715*, and *HTRA1* genes were associated with exudative AMD in our independent cohort of northern Chinese patients.

## Methods

### Patients and control individuals

Two independent groups of Chinese individuals, including patients with exudative AMD and control subjects, were recruited during outpatient visits to the Beijing Tongren Hospital, Capital Medical University, China. All cases and controls were unrelated native Chinese from the greater Beijing area, northern China. The study protocol was approved by the Ethics Committee of Beijing Tongren Hospital. Informed consent was obtained from all participants, and the procedures used conformed to the tenets of the Declaration of Helsinki for research involving human subjects. A total of 253 individuals, including 121 cases with exudative AMD (71 males and 50 females) and 132 control subjects (68 males and 64 females) participated in this study. The age range is 50 to 90 years old with mean 66.0 and standard deviation (SD) 8.4 in cases, and 50 to 84 years old with mean 66.1 and SD 6.6 in controls.

### Ophthalmic examination

All participants received a standard ophthalmic examination, including visual acuity measurement, slit-lamp biomicroscopy, and dilated fundus examination that was performed by a retinal specialist. All AMD patients had fluorescein as well as indocyanine green fundus angiography. The diagnosis of exudative AMD was based on ophthalmoscopic and fluorescein angiographic findings. Inclusion criteria for patients were as follows: 1) age of 50 years or older, 2) presence of macular lesions (choroidal neovascular membrane, subretinal hemorrhage, RPE detachment, and fibrovascular disciform scars) in one or both eyes; and 3) macular lesions not associated with other eye diseases, such as degenerative myopia, angioid streaks, or any other retinal/choroidal diseases. Controls were confirmed not to have clinical evidence of early or late AMD in both eyes and not to have any other eye diseases aside from mild age-related cataracts. Subjects with severe cataracts were excluded from the study.

### DNA extraction and polymerase chain reaction

Blood samples were collected from all participants and stored at -80 °C before DNA extraction. Genomic DNA was extracted from the peripheral venous blood for each participant using a commercially available genomic DNA extraction and purification kit (TIANamp Swab DNA Kit; Tiangen Biotech, Beijing, China) according to the manufacturer’s protocol. Genotyping was performed using a method of polymerase chain reaction (PCR) followed by allele-specific restriction enzyme digestion and direct sequencing. The primer sequences used in this study are given in Table 1. PCR reactions were performed using a DNA thermocycler (Eppendorf, Hamburg, Germany) in a 25 µl mixture containing 2.5 µl 10X buffer (25 mM/L MgCl_2_ ), 25 µM each of dNTP, 1 pmol of each primer, 0.5 units *Taq* DNA polymerase, and 50 ng genomic DNA. Amplification of *HTRA1* rs11200638 was performed by adding 5% dimethyl sulfoxide (DMSO) to the PCR reaction mixture. Samples were denaturing at 94 °C for 5 min followed by 35 cycles under the following conditions: denature at 94 °C for 30 s, annealing at 56 °C for 30 s, and extension at 72 °C for 45 s. The final extension step was lengthened to 5 min. Aliquots of amplified uncut products were resolved by electrophoresis in 2% (w/v) agarose gels with 0.5 µg/ml ethidium bromide and visualized under ultraviolet light. Samples were then used for allele-specific restriction enzyme digestion. Some products were also used for direct sequencing.

**Table 1:** Primers used in polymerase chain reaction amplification and sequencing

**Gene**	**SNP**	**Primer sequence (5’→3’)**
*CFH*	rs1061170	F: TCATTGTTATGGTCCTTAGGAAA
		R: GGAGTAGGAGACCAGCCATT
*LOC387715*	rs10490924	F: TACCCAGGACCGATGGTAAC
		R: GAGGAAGGCTGAATTGCCTA
*HTRA1*	rs11200638	F: ATGCCACCCACAACAACTTT
		R: CGCGTCCTTCAAACTAATGG

The primer sequences used in polymerase chain reaction (PCR) amplification and sequencing of the single-nucleotide polymorphisms (SNPs) in complement factor H (*CFH*), *LOC387715*, and *HTRA1* genes.

### Restriction digestion and direct sequencing

**Figure 1:**
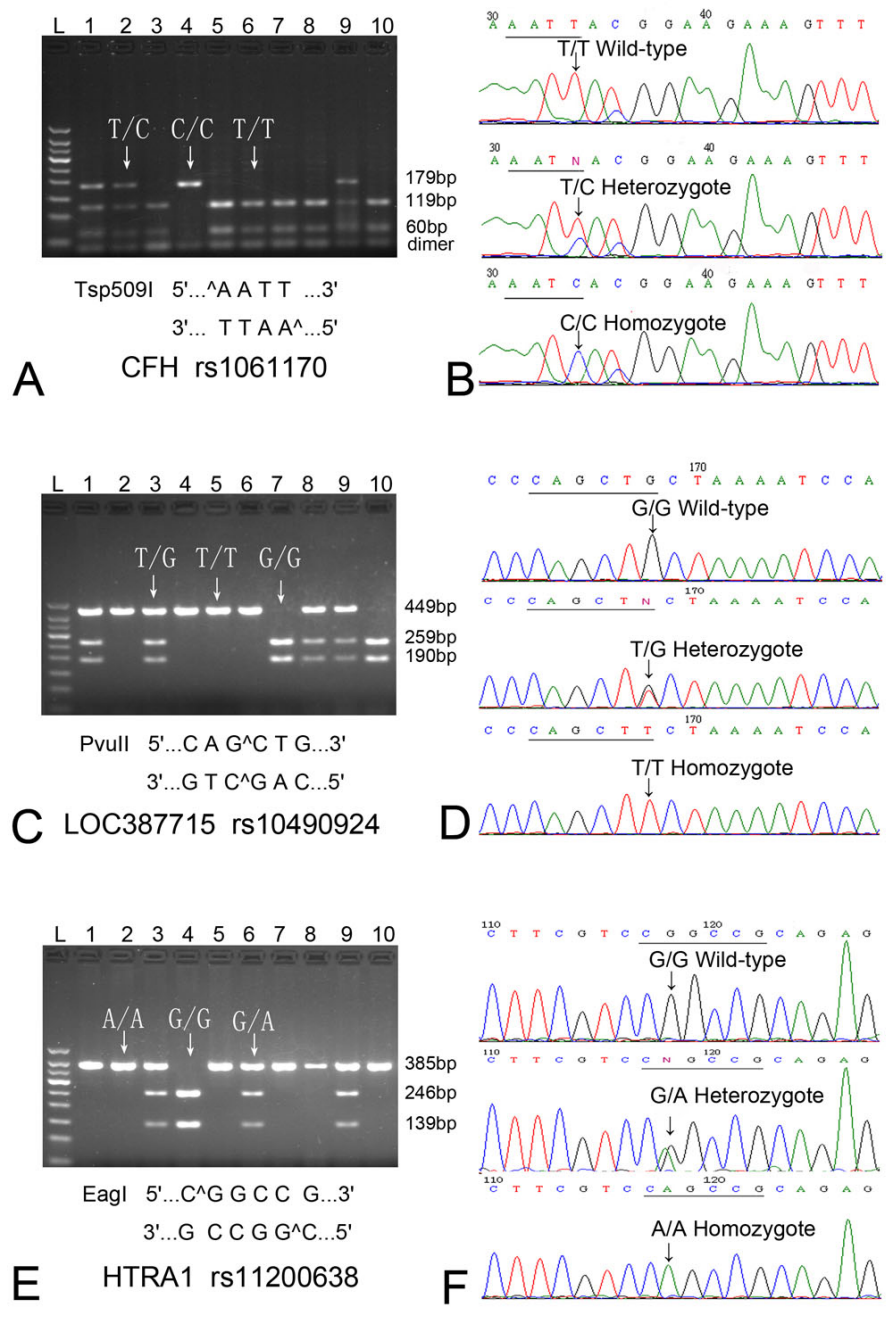
Ethidium bromide-stained 2% agarose gels and direct sequencing, showing the PCR-restriction fragment length polymorphism and corresponding sequence chromatogram. **A:** Restriction analysis for complement factor H (*CFH*) Y402H polymorphism resulted in digestible fragment (T/T), undigestible fragment (C/C), and heterozygote (T/C). **B:** Direct sequencing of the PCR products confirmed the restriction patterns for CFH Y402H. **C:** Restriction analysis for *LOC387715* rs10490924 resulted in digestible fragment (G/G), undigestible fragment (T/T), and heterozygote (T/G). **D:** Sequencing confirmed the restriction patterns for *LOC387715* rs10490924. **E:** Restriction analysis for *HTRA1* rs11200638 resulted in digestible fragment (G/G), undigestible fragment (A/A), and heterozygote (G/A). **F:** Sequencing confirmed the restriction patterns for *HTRA1* rs11200638.

The amplified products were analyzed by restriction enzyme digestion according to the manufacturer’s protocol (New England Biolabs, Ipswich, MA). Enzymes used in this study and the lengths of various restriction fragments are given in Figure 1. Restriction digestion was performed at either 65 °C for 3 h (Tsp509I for *CFH* Y402H) or 37 °C for 3 h (PvuII for *LOC387715* and EagI for *HTRA1*). Samples were electrophoresed on a 2% (w/v) agarose gel with 0.5 µg/ml ethidium bromide. Images of the gel were taken with a Molecular Imager Gel Doc XR System (Bio-Rad, Hercules, CA). Genotypes were determined based on the restriction patterns and were further confirmed by direct sequencing of the PCR products using an automatic ABI 3730XL DNA Analyzer (Applied Biosystems, Foster City, CA) in a selected subset of subjects (Figure 1).

### Statistical analysis

Hardy Weinberg equilibrium (HWE) χ^2^ values in cases and controls were calculated to identify possible genotyping errors. Linkage disequilibrium (LD) was measured by the D' value using statistical software Haploview version 4.0 [28]. Numerical data were examined by Student *t* test. Genotypes and allele frequencies between cases and controls were compared using the χ^2^ test. Odd ratios (OR) and 95% confidence intervals (CI) were calculated according to the Woolf equation [29]. A p-value of <0.05 was considered statistically significant.

## Results

Case–control samples were composed of 121 unrelated patients with exudative AMD and 132 healthy control individuals (Table 2). The mean ages were 66.0±8.4 for AMD patients and 66.1±6.6 years for healthy controls (p=0.94, Student *t* test). Percentage of males was 58.7% in the AMD group and 51.5% in the control group (p=0.25, χ^2^ test).

**Table 2:** Age and sex of patients with exudative AMD and control subjects

**Sample**	**AMD cases (n=121)**	**Controls (n=132)**	**All subjects (n=253)**	**p value**
Age (year)	66.0±8.4	66.1±6.6	66.0±7.5	0.94
Sex				
Male	71	68	139	
Female	50	64	114	0.25

The case-control samples were composed of 121 unrelated patients with exudative age-related macular degeneration (AMD) and 132 control individuals. No statistical difference was observed between AMD patients and controls groups for age (p=0.94, Student t test) or gender (p=0.25, χ^2^ test).

Genotypes were determined successfully by restriction enzyme digestion in all participants for SNPs rs1061170:T>C in *CFH* (Y402H), rs10490924:G>T in *LOC387715* (A69S), and rs11200638:G>A in the promoter region of *HTRA1* (Table 3). Genotypes were confirmed by direct sequencing in 82 randomly selected subjects, including 40 case participants and 42 control individuals, and the sequencing data were consistent with the restriction enzyme digestion results in all studied subjects. Genotype distributions for SNPs at gene loci *CFH*, *LOC387715*, and *HTRA1* were in Hardy–Weinberg equilibrium in either AMD cases or control subjects (p-value ≥ 0.283).

**Table 3:** Genotype and allele frequencies in cases with exudative AMD and controls

**Genotypes**	**AMD cases (n=121)**	**Controls (n=132)**	**χ^2^**	**p value**	**OR (95%CI)**
*CFH*rs1061170:T>C (*Y402H*)					
Genotype					
TT	97 (80.2%)	111 (84.3%)			1.0 (reference)
TC	23 (19.0%)	21 (15.7%)	0.463	0.496	1.25 (0.65-2.40)
CC	1 ( 0.8%)	0 ( 0)	-	-	-
	H-W p=1.0	H-W p=0.843			
Allele					
T	217 (89.7%)	243 (92.0%)			1.0 (reference)
C	25 (10.3%)	21 ( 8.0%)	0.863	0.353	1.33 (0.73-2.45)
*LOC387715*rs10490924:G>T (A69S)					
Genotype					
GG	18 (14.9%)	40 (30.3%)			1.0 (reference)
GT	49 (40.5%)	70 (53.0%)			1.56 (0.80-3.03)
TT	54 (44.6%)	22 (16.7%)	25.094	<0.001	5.45 (2.59-11.49)
	H-W p=0.283	H-W p=0.480			
Allele					
G	85 (35.1%)	150 (56.8%)			1.0 (reference)
T	157 (64.9%)	114 (43.2%)	23.89	<0.001	2.43 (1.70-3.48)
*HTRA1*rs11200638:G>A					
Genotype					
GG	13 (10.7%)	44 (33.3%)			1.0 (reference)
GA	52 (43.0%)	64 (48.5%)			2.75 (1.34-5.64)
AA	56 (46.3%)	24 (18.2%)	30.48	<0.001	7.90 (3.61-17.26)
	H-W p=0.980	H-W p=1.0			
Allele					
G	78 (32.2%)	152 (57.6%)			1.0 (reference)
A	164 (67.8%)	112 (42.4%)	32.711	<0.001	2.82 (1.96-4.06)

This table summarizes the genotype and allele frequencies for the single-nucleotide polymorphisms (SNPs) in complement factor H (*CFH*), *LOC387715*, and *HTRA1* genes among patients with exudative age-related macular degeneration (AMD) and control subjects. Genotype distributions for SNPs were in Hardy-Weinberg (H-W) equilibrium in either cases or controls. The p-value represents comparison or level of risk significance between AMD cases and controls. Adjustment for multiple comparisons was made using Bonferroni method, which did not change the significant levels for all three SNPs. OR indicates odds ratio and CI refers to confidence interval.

Between case participants and controls, the *CFH* variant Y402H was not associated with exudative AMD (Table 3). The frequencies for the risk C allele at Y402H were 10.3% in AMD cases and 8.0% in controls (p=0.353, χ^2^ test) in the study population. No significant difference between the CFH genotypes in AMD group and control group was observed (p=0.496, χ^2^ test). Compared to the wild-type homozygous TT genotype, the OR for heterozygous TC genotype was 1.25 (95% CI; 0.65–2.40). The OR for homozygous CC genotype was not calculated since the CC homozygote was identified only in one case in this Chinese cohort.

A strong association with exudative AMD was detected for SNP rs10490924:G>T in *LOC387715* (Table 3). The risk T allele frequencies were 64.9% for AMD cases and 43.2% for controls (p<0.001, χ^2^ test). Genotype distributions between AMD cases and controls were statistically significantly different (p<0.001, χ^2^ test). Compared to wild-type GG genotype, the OR for the risk of AMD was 1.56 (95% CI; 0.80–3.03) for the heterozygous GT genotype and 5.45 (95% CI; 2.59–11.49) for the homozygous TT genotype.

Similarly, the SNP rs11200638:G>A in the promoter of *HTRA1* was significantly associated with exudative AMD (Table 3). Frequencies of the risk A allele were 67.8% for AMD cases and 42.4% for controls (p<0.001, χ^2^ test). Genotypes of this HTRA1 promoter polymorphism between AMD cases and controls were statistically significantly different (p<0.001, χ^2^ test). The OR was 2.75 (95% CI; 1.34–5.64) for the heterozygous GA genotype and 7.90 (95% CI; 3.61–17.26) for the homozygous AA genotype, compared to the wild-type GG genotype.

The *LOC387715* rs10490924 and *HTRA1* rs11200638 genotype combination frequencies in patients with exudative AMD and control subjects are shown in Table 4. An OR of 7.94 (95% CI; 3.49–18.04) was obtained for carriers with combined TT genotype of *LOC387715* and AA genotype of *HTRA1* (Table 4). *LOC387715* rs10490924 and *HTRA1* rs11200638 were in high LD in cases (D’=0.98, LOD=38.09, r^2^=0.843) and in controls (D’=0.869, LOD=32.55, r^2^=0.732).

**Table 4:** *LOC387715* rs10490924 and *HTRA1* rs11200638 genotype combination frequencies in patients with exudative AMD and control subjects

		***HTRA1***		
		GG	AG	AA
**AMD cases**	*LOC387715* GG	13 (10.7%)	5 ( 4.1%)	0
	TG	0	46 (38.0%)	3 ( 2.5%)
	TT	0	1 ( 0.8%)	53 (43.8%)
**Control subjects**	*LOC387715* GG	37 (28.0%)	4 ( 3.0%)	0
	TG	7 ( 5.3%)	58 (43.9%)	5 ( 3.8%)
	TT	0	2 ( 1.5%)	19 (14.4%)
**OR (95%CI)**	*LOC387715* GG	1.0 (reference)	3.56 (0.83-15.30)	-
	TG	-	2.26 (1.08-4.74)	1.71 (0.36-8.16)
	TT	-	1.42 (0.12-17.03)	7.94 (3.49-18.04)

This table shows the two-locus (*LOC387715* and *HTRA1*) genotype combination frequencies in patients with exudative age-related macular degeneration (AMD) and control subjects. The combined presence of the risk *LOC387715* TT genotype and *HTRA1* AA genotype was more commonly found in cases than in controls and represented an increased risk for AMD. OR indicates odds ratio and CI refers to confidence interval.

## Discussion

The missense polymorphism Y402H in *CFH* (rs1061170) has been identified as a common nonsynonymous variant and a major genetic risk factor for AMD development in Caucasian populations. Frequencies of the risk C allele in Caucasians were between 61%–94% in AMD and 34%–46% in controls [15-21]. In contrast, in our northern Chinese study population, the C allele was low in frequency (10.3% in cases and 8.0% in controls) and was not associated with exudative AMD. Other research groups in Hong Kong and Taiwan also reported low frequencies of the risk C allele in their study populations [30,31]. In Hong Kong Chinese, the frequency of the risk C allele was 5.8% in AMD cases and 3.9% in controls [30]. In Taiwan Chinese, the frequency of the risk C allele was 11.3% in AMD cases and 2.8% in controls [31]. Their conclusions about the association of Y402H with exudative AMD, however, were controversial. Chen et al. [30] found no association between the Y402H polymorphism and exudative AMD in Hong Kong Chinese population as we did in our study. Lau et al. [31] concluded, however, that Y402H was significantly associated with exudative AMD in Taiwan Chinese. This disparity may be due to sampling bias, difference in inclusion criteria, demographic factors, or variations in Chinese subpopulations. Nonetheless, the genetic attributable risk of Y402H in Chinese populations could not be substantial because the allele frequency was low based on the current study and the data of others [30,31]. Several case-control studies in Japanese populations have shown that the Y402H variant was also at a low frequency and not associated with AMD [32-35]. Dramatic differences may exist in the allele frequencies of individual SNPs across populations [36].

*CFH* is involved in the regulation of the alternative complement pathway. Complement components have previously been found in drusen, an inflammatory deposit between retinal pigment epithelium and neuron-sensory retina, suggesting dysregulation of complement activation may be involved in AMD development [37]. Recently, Laine et al. [38] reported that the binding of the *CFH* Y402H variant to C-reactive protein was strongly reduced compared to the wild-type, indicating that the association of the *CFH* Y402H with AMD could be due to reduced clearance of cellular debris and increased local inflammation. Magnusson et al. [39] demonstrated that the Y402H variant confers a similar risk of soft drusen and advanced forms of AMD, and hypothesized that the Y402H variant is a major risk factor for soft drusen formation but additional genetic as well as environmental factors may affect progression to exudative AMD. In studies conducted in the Chinese population, however, drusen is less frequently observed and the prevalence of late-stage AMD has been found to be lower when compared with Caucasians [40,41]. The low frequency of Y402H variant and its genetic susceptibility to AMD in our cohort as presented in this study may correlate with the epidemiological features of AMD in the Chinese population. Similar phenotypes of AMD and genetic correlation with *CFH* Y402H variant have been reported in Japanese populations [32-35,42,43]. A relationship between ethnic differences in disease-susceptible genetic variants and ethnic diversity in phenotypes has also been suggested in other diseases [44-46].

In contrast to *CFH* Y402H variant, our data demonstrate strong associations for the risk of exudative AMD with *LOC387715* and *HTRA1* variants. Consistent with previous published findings for the Hong Kong Chinese [26] and Caucasian populations [27,47-49], our study shows that AMD risk is higher with the T allele of *LOC387715* rs10490924 or the A allele of rs11200638 in the promoter region of *HTRA1*. This finding is also consistent with those of Japanese studies published recently [50-52]. The data presented here support the hypothesis that the *LOC387715* and *HTRA1* genes associate with susceptibility to AMD development across diverse ethnicities. In this study, *LOC387715* rs10490924 and *HTRA1* rs11200638 showed similar significance levels. ORs for exudative AMD were 1.56 (95%CI, 0.80-3.03) for the heterozygous GT genotype and 5.45 (95%CI, 2.59-11.49) for the homozygous TT genotypes of *LOC387715* rs10490924, when compared to the wild GG genotype. ORs for exudative AMD with heterozygous GA genotype and homozygous AA genotype of rs11200638 in the promoter of *HTRA1* were 2.75 (95% CI; 1.34–5.64) and 7.90 (95% CI; 3.61–17.26), respectively, when compared to the wild-type GG genotype. Individuals with both TT genotype of *LOC387715* and the AA genotype of *HTRA1* associated with an OR of 7.94 (95% CI; 3.49–18.04), similar to the risk conferred by rs10490924 or rs11200638 alone. Based on the likelihood ratio test, no interaction or combined effect was evident between the two SNPs: rs10490924 and rs11200638.

*LOC387715* and *HTRA1* are both located on the chromosome 10q26 region, which is one of the major chromosomal regions identified for AMD susceptibility*.* *LOC387715* encodes a hypothetical protein of unknown function and is highly expressed in placental tissue, but its expression in the human retina is weak [24]. The risk T allele of SNP rs10490924 maps to exon 1 of the hypothetical *LOC387715* gene and changes putative amino acid 69 from alanine to serine (A69S). *HTRA1* encodes a heat shock serine protease and is expressed in the mouse and human retina [26]. SNP rs11200638 resides in the promoter of *HTRA1* and is approximately 6.1 kb downstream of the *LOC387715* rs10490924. Because only a single cDNA sequence of *LOC387715* has been found, DeWan and co-authors [26] hypothesized that SNP rs10490924 is a surrogate marker that is correlated, or is in LD, with the putative AMD disease-causing variant. They thus concluded that *HTRA1* is a major risk factor for exudative AMD. Contrary to the reports of DeWan et al. and others [26,27], a study by Kanda et al. [53] showed that *LOC387715*, but not *HTRA1*, represents a major susceptibility variant for AMD at 10q26. In their study, Kanda and coauthors [53] showed that SNP rs11200638 has no significant impact on *HTRA1* promoter activity in three different cell lines, and *HTRA1* mRNA expression exhibits no significantly different change between control and AMD retinas. However, it has been demonstrated that *LOC387715* mRNA is detected in the human retina and various cell lines and encodes a 12 kDa protein, which localizes to the mitochondrial outer membrane when expressed in mammalian cells [53]. Kanda and co-authors [53] therefore proposed that the A69S change in the LOC387715 protein affects its presumptive function in mitochondria and enhances the susceptibility to aging-associated degeneration of macular photoreceptors. In agreement with previous studies, we show in this study that *LOC387715* rs10490924 and *HTRA1* rs11200638 are in high LD. Further studies are needed to clarify whether *LOC387715* rs10490924 and *HTRA1* rs11200638 are only in LD or are causative factors for AMD.

In summary, our data demonstrated that *LOC387715* and *HTRA1*, but not the *CFH* Y402H polymorphism, conferred significantly increased risk for exudative AMD in a northern Chinese population. Replication of association studies in diverse ethnic groups worldwide may provide a better appreciation of the genetic contributions in AMD pathogenesis. Further studies would be needed to determine the identity of causal variant and to evaluate the possible mechanisms through which the variant influences the disease susceptibility.
